# Analyzing University of Virginia Health publications using open data, Python, and Streamlit

**DOI:** 10.5195/jmla.2021.1360

**Published:** 2021-10-01

**Authors:** Anson Parker, Abbey Heflin, Lucy Carr Jones

**Affiliations:** 1 adp6j@virginia.edu, Web Developer, University of Virginia Claude Moore Health Sciences Library, Charlottesville, VA; 2 aeh6m@virginia.edu, Manager for Collections and Resource Strategy, University of Virginia Claude Moore Health Sciences Library, Charlottesville, VA; 3 lgc3t@virginia.edu, Library Assistant, University of Virginia Claude Moore Health Sciences Library, Charlottesville, VA

## Abstract

As part of a larger project to understand the publishing choices of UVA Health authors and support open access publishing, a team from the Claude Moore Health Sciences Library analyzed an open data set from Europe PMC, which includes metadata from PubMed records. We used the Europe PMC REST API to search for articles published in 2017–2020 with “University of Virginia” in the author affiliation field. Subsequently, we parsed the JSON metadata in Python and used Streamlit to create a data visualization from our public GitHub repository. At present, this shows the relative proportions of open access versus subscription-only articles published by UVA Health authors. Although subscription services like Web of Science, Scopus, and Dimensions allow users to do similar analyses, we believe this is a novel approach to doing this type of bibliometric research with open data and open source tools.

Spurred by ongoing discussions about the attitude of UVA Health authors toward open access and open science, a team at the University of Virginia (UVA) Claude Moore Health Sciences Library decided to examine where UVA Health faculty, staff, and students were publishing and how many of those articles were open access. The team decided to create an online dashboard to visualize the information for use in internal discussions about collection development as well as conversations with external stakeholders about open access. To minimize the need for manual updates in the future, the team decided to use an application programming interface (API) as the data source. APIs are how one piece of software communicates with another, and so using an API as the data source rather than a spreadsheet means that the dashboard can continually be updated automatically. Although UVA has institutional subscriptions to commercial software that measures research impact, the license terms for those products usually restrict how their data can be shared, which would limit the possibilities for future development of the dashboard. To align with the project's goal of encouraging open behaviors, the team decided to share the code openly and use only open data sources so that anyone could replicate or adapt the dashboard to study their own institution's publications.

The first step was researching what open data sources on biomedical publications were available. To restrict the search to only UVA Health authors, an API that included institutional affiliation for all authors was necessary. The team initially considered using the Cold Spring Harbor API (indexing medRxiv and bioRxiv) to study preprints, but discarded that option after realizing that it only included institutional affiliation for the corresponding author. The Directory of Open Access Journals data was also considered, but a search for University of Virginia in the “affiliation” field returned only a few thousand results, suggesting that the data was incomplete. The Crossref API, which indexes journal articles as well as preprints was another option, but Crossref's broad coverage of articles from all scholarly disciplines meant that it would require additional steps to refine the data set to reflect biomedical publications only. The Europe PMC API proved to be the best data source for this project as it focuses on biomedical publications, and the core metadata option for their API includes the institutional affiliation for all authors separated into individual fields. A search for “UVA” did not yield any relevant results while a search for “University of Virginia” yielded tens of thousands of relevant results, suggesting that the vast majority of articles used that wording for the affiliation of UVA Health authors.

After deciding on a data source, the team needed to find a way to process and visualize the data. Streamlit is a Python library that can be used to create interactive web applications using simple code. To take advantage of Streamlit's free sharing service, the code must be housed in a public GitHub repository. After connecting the GitHub repository to the Streamlit account, it took some trial and error to get the full results from the Europe PMC query to load. Helpfully, Europe PMC operates a listserv for developers, and a Europe PMC staff member replied to the team's email with a piece of code that enabled the full data set to load properly. Another stumbling block was the dashboard's long load time, but this was greatly improved by implementing Streamlit's built-in caching feature, as suggested by users on the company's community discussion board and Streamlit employees on Twitter. The dashboard now shows the proportion of open access versus subscription-only articles published by UVA-affiliated authors indexed in Europe PMC from 2017 to the present. The team also added an exploratory data analysis (EDA) widget made using the Python library Sweetviz to provide a quick visualization of the dataset and help uncover potential areas for future analysis.

